# Supplementary material for: Clinical outcomes and management of tibial plateau fractures in Ethiopia: A prospective cohort study
Source: PLoS One. 2025 May 23;20(5):e0323956. doi: 10.1371/journal.pone.0323956 (PMC12101707; doi:10.1371/journal.pone.0323956)
Supplement: S1 File — (PDF) [file pone.0323956.s001.pdf]

ባሕር ዳር ዩኒቨርሲቲ
ሕክምና እና ጤና ሳይንስ ኮሌጅ
የስነ - ምግባር ገምጋሚ ቦርድ
ባሕር ዳር፣ኢትዮጵያ

Bahir Dar University
College of Medicine and Health Sciences
Institutional Review Board
Bahir Dar, Ethiopia

✉ ———— ☏ 058 89 99 275 /058 89 99271
Fax: ———— Website: www.bdu.edu.et/cmhs

E-mail :cmhs@bdu.edu.et
Facebook: College of Medicine and Health Sciences, Bahir Dar University, Ethiopia

IRB's Decision (Extension)

Meeting No.: Expedit 2018
Protocol number: 433/2018

Date: January 06, 2018
assigned No: 003

Protocol Title: - Clinical out come and mangment of tibial plateau fracture			
**Principal investigator:**	Adugnaw Bogale		
Co-investigators			
**Institute:**	College of Medicine and Health Sciences, Bahir Dar University		
**Elements Reviewed (CMHS/IRB 01 - 008)**		☑ **Attached**	☐ Not attached
0Review of Revised Application ☐ Yes ☑ No		Date of Previous review:	
Decision of the meeting:	☑ **Approved**	☐ Approved with Recommendation	
	☐ Resubmission	☐ Disapproved	

Elements approved:

1. Protocol Version No.: **01**
2. Protocol Version Date October 06, **2017**
3. Informed Consent Version: **01**
4. Informed Consent Version Date October 06,**2017**

Obligations of the PI:

- ✓ Comply with Standard National and International Ethical Guidelines
- ✓ All Amendments and Changes made in the Protocol and Consent Needs IRB Approval
- ✓ Report SAE within 10 days of the event
- ✓ End of the study, including manuscript and thesis works should be reported to IRB

To NRERC ✓ ☐

Institutional Review Board (IRB) Approval: Period from *01/19/2018* to 06/*03/2022*

Follow-up Report Expected in:

One Year ______ Two Year ______ Three Year ______ Four Year ✓

Chairperson, IRB - Mulusew Andualem(PhD)

Signature ______

Date: January 06, 2018
