# Supplementary material for: Clinical outcomes and management of tibial plateau fractures in Ethiopia: A prospective cohort study
Source: PLoS One. 2025 May 23;20(5):e0323956. doi: 10.1371/journal.pone.0323956 (PMC12101707; doi:10.1371/journal.pone.0323956)
Supplement: Attachment — Submitted filename: Reviewer 2 .docx [file pone.0323956.s005.docx]

Reviewer 2:

The authors describe clinical outcomes one year after tibial plateau fractues in a single center hostpital in Ethiopia. There are only few studies about this topic in Africa and therefore the topic is very important and novel to report.

Thank you!

1-In the reviewed manuscript line numbering and the tables are missing. This makes sound reviewing difficult. Nevertheless there are a few comments already implementable by the authors to improve the quality of the study at hand.

Answer, Accept and corrected.

2- The newest reference is from 2019. There is much more literature about these fractures available, for example:

https://pubmed.ncbi.nlm.nih.gov/?term=Tibial+Plateau+Fractures+in+africa

3- The statistical testing is not done properly. Data need to be tested for normal distribution and thereafter are tested with the tests accordingly. Furthermore, why was mulivariate logistic regression performed? The power of the data sample is probably not allowing this test. Univariate analysis is of course possible. Simple t-test, chi square, fisher’s exact test and Mann-Whitney U test respectively should be accurate for the data sample.

Answer: Our study included a total population of 191 participants, which we considered to be sufficiently large for the analyses performed. We initially conducted bivariate analysis for each independent variable and the outcome variable (functional outcome). Based on the statistical significance observed in the bivariate analysis, we proceeded with multivariate analysis to assess the associations between these variables. Additionally, Spearman's correlation was used to evaluate relationships between variables, such as immobilization duration and functional outcomes. We chose to perform multivariate logistic regression due to the significance observed in the bivariate analysis, and aimed to explore these associations more comprehensively. While univariate analyses, including t-tests, chi-square, Fisher's exact test, and Mann-Whitney U test, would also be appropriate for this dataset, we believed that the sample size was adequate to support the analyses conducted.

3- Please adhere to the Strobe Checklist for reporting of your study

<https://www.strobe-statement.org/checklists/>

Answer; accepted and adhered

4- I further recommend defining a primary outcome: For example functional follow up at one year and define all other values as secondary outcomes. Therewith the very nicely conducted study could further gain value.

Answer: Accept and Corrected. In methodology part.
